# Correction: Quantification of speech and synchrony in the conversation of adults with autism spectrum disorder

**DOI:** 10.1371/journal.pone.0227387

**Published:** 2020-01-17

**Authors:** The PLOS ONE Staff

In the “Speech feature processing” subsection of the Methods, a bracket is missing in the beginning of the sixth equation. The publisher apologizes for the error. Please view the complete, correct equation here:

$$\Gamma_{B}\left[\text{k}\right]=\left\{i\;|\frac{n}{2}k\leq i\leq \frac{n}{2}\left(2k+1\right)\wedge x\left[i\right]\;\text{is}\;\text{calculated}\;\text{at}\;\text{the}\;i\text{th}\;\text{frame}\right\}$$

In the PDF version of the article, the pound symbol is missing from the first sentence after the sixth equation in the “Speech feature processing” subsection of the Methods. The publisher apologizes for the error. The correct sentence is: Note that #ΓB[k] equals the numbers of the frames included in IPUs in the case of intensity, whereas #ΓB[k] is the number of voiced frames in that block in the case of F_0_.

In Fig 5, the x-axis labels are incorrectly placed. There are also a number of errors in the caption for Fig 5, “Scatter plots between the features that have significant correlations with the reciprocity score.” Please see the complete, correct Fig 5 and caption here.

**Fig 5:**
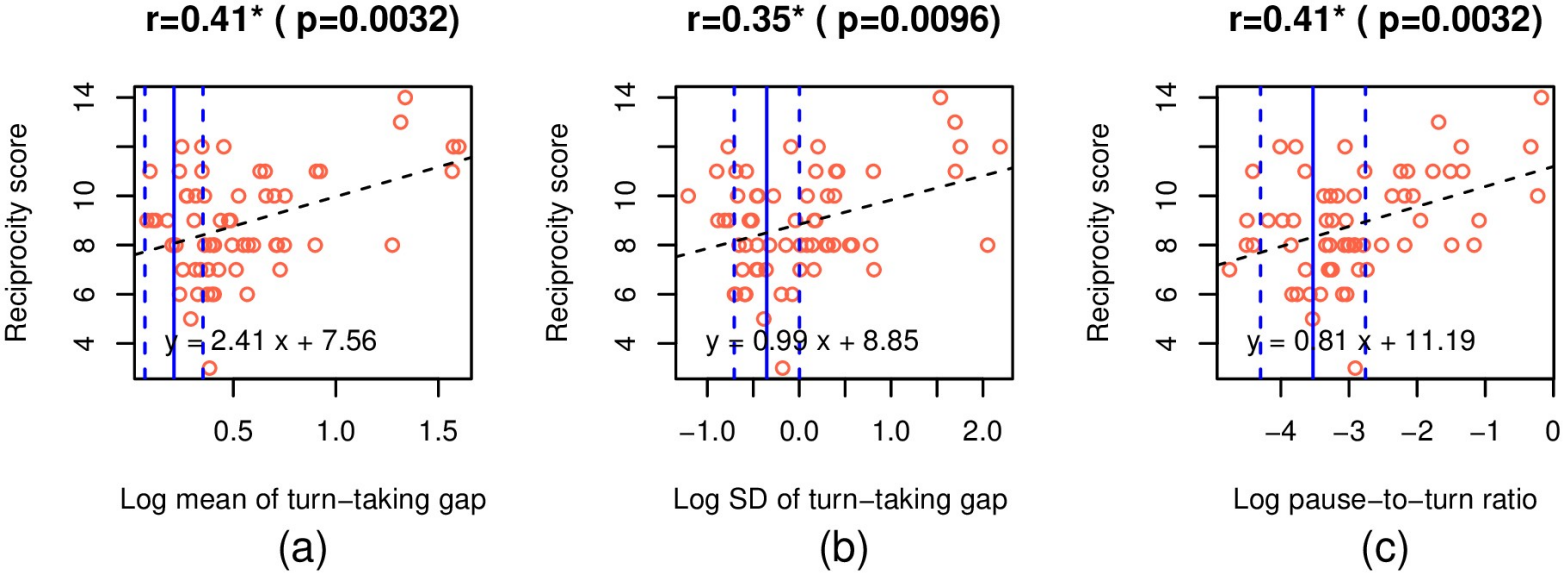
Scatter plots between the features that have significant correlations with the reciprocity score. The black dotted line represents a regression line. The blue solid and dashed lines represent the mean and mean ± SD of the feature among the participants with TD, respectively. Reciprocity score of the individuals with ASD were positively correlated with the mean and SD of turn-taking gap and log of pause-to-turn ratio. At the same time, the distributions of the ASD and TD groups overlapped in the area between −5 and −2. Similar overlaps were observed in mean and log of SD of turn-taking gap.
