# Supplementary figures and images for: Quantification of speech and synchrony in the conversation of adults with autism spectrum disorder
Source: PLoS One. 2019 Dec 5;14(12):e0225377. doi: 10.1371/journal.pone.0225377 (PMC6894781; doi:10.1371/journal.pone.0225377)

a)

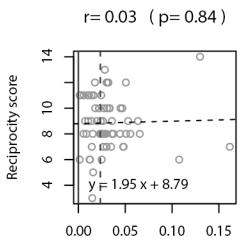

(a-1)

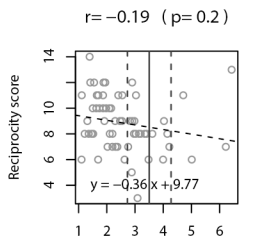

(a-2)

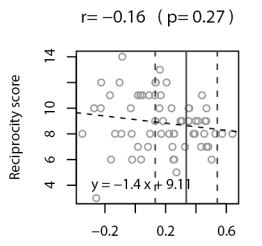

(a-3)

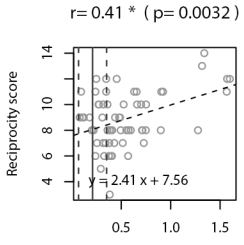

(a-4)

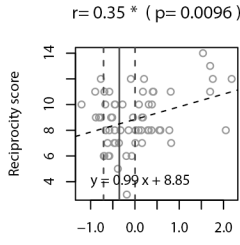

(a-5)

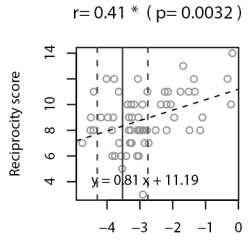

(a-6)

b)

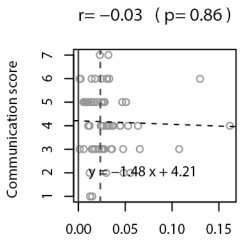

(b-1)

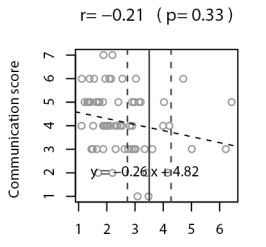

(b-2)

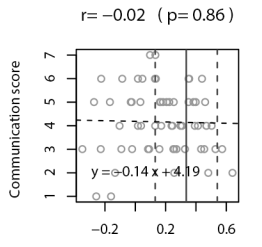

(b-3)

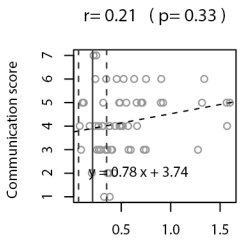

(b-4)

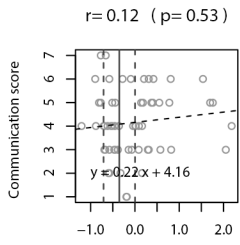

(b-5)

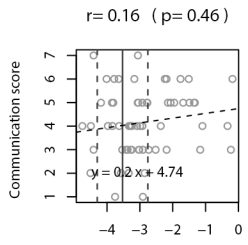

(b-6)

c)

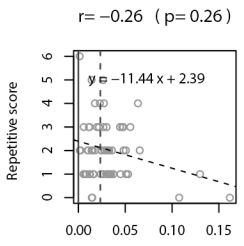

(c-1)

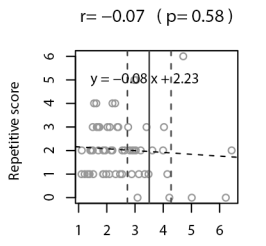

(c-2)

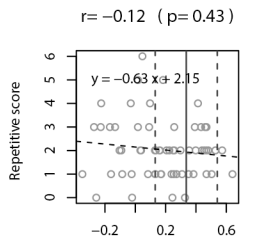

(c-3)

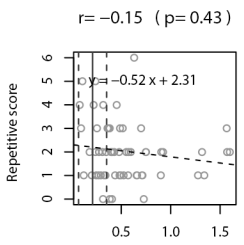

(c-4)

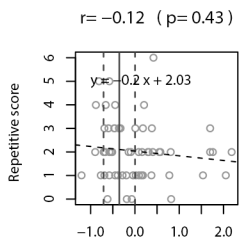

(c-5)

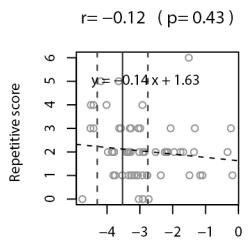

(c-6)

Supplement: S1 Fig — The (r) and p-value for correlation coefficient is shown in the top of each plot. (PDF) [file pone.0225377.s001.pdf]
